# Therapeutic Potential of Jasmonic Acid and Its Derivatives

**DOI:** 10.3390/ijms22168437

**Published:** 2021-08-05

**Authors:** Iwona Jarocka-Karpowicz, Agnieszka Markowska

**Affiliations:** Department of Analytical Chemistry, Medical University of Bialystok, 15-022 Bialystok, Poland; agnieszka.markowska@umb.edu.pl

**Keywords:** jasmonic acid, methyl jasmonate, anti-inflammation, anti-cancer, anti-aging

## Abstract

A modern method of therapeutic use of natural compounds that would protect the body are jasmonates. The main representatives of jasmonate compounds include jasmonic acid and its derivatives, mainly methyl jasmonate. Extracts from plants rich in jasmonic compounds show a broad spectrum of activity, i.e., anti-cancer, anti-inflammatory and cosmetic. Studies of the biological activity of jasmonic acid and its derivatives in mammals are based on their structural similarity to prostaglandins and the compounds can be used as natural therapeutics for inflammation. Jasmonates also constitute a potential group of anti-cancer drugs that can be used alone or in combination with other known chemotherapeutic agents. Moreover, due to their ability to stimulate exfoliation of the epidermis, remove discoloration, regulate the function of the sebaceous glands and reduce the visible signs of aging, they are considered for possible use in cosmetics and dermatology. The paper presents a review of literature data on the biological activity of jasmonates that may be helpful in treatment and prevention.

## 1. Jasmonate Compounds in Plants

Jasmonates are lipid derivatives (cyclic derivatives of unsaturated fatty acids) that belong to the group of plant growth regulators, which do not have a complex chemical structure [1]. The best known compounds belonging to the group are jasmonic acid (JA) and its methyl ester–methyl jasmonate (MJ) [2]. Jasmonic acid was first isolated from filtrates of the fungus *Lasiodiplodia theobromae* [3]. Its methyl derivative, however, was the first compound from the large group of jasmonates isolated from the essential oils of *Jasminum grandiflorurm* [4] and *Rosmarinum officinalis* [5].

The presence of jasmon compounds has been confirmed in almost all types of tissues of higher plants, i.e., flowering plants, bryophytes, and ferns. They are present, among others, in stems (combinations with amino acids), roots, tubers, leaves (combinations with amino acids; isoleucine or valine), flowers (conjugates with phenylalanine, tryptophan, and tyrosine), fruits (conjugates with isoleucine), and flower pollen [6]. Jasmonates are also components of essential oils and give fragrance to many flowers (e.g., jasmine) and fruits (e.g., apples). Jasmonic acid, as the basic representative of this group of compounds, is present in plants more often than its methyl ester, with the exception of *Malus sylvestris* fruits, where both compounds occur simultaneously. The presence of jasmonates has also been studied in lower plants (e.g., algae–*Chorella*) and fungi (*Gibberella fujikuroi* and *Botryodiplodia theobromae*). Unfortunately, previous studies have not revealed which plant parts are responsible for their synthesis [7].

**Figure 1:**
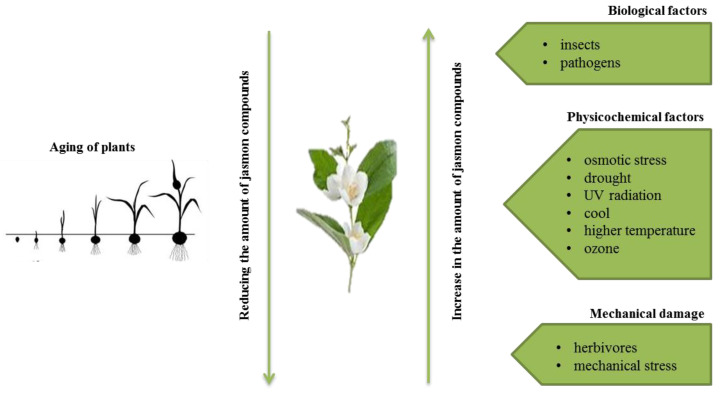
Factors influencing the changes in the levels of jasmonate compounds in the plant.

Depending on the type, species, and age of the plant, the content of jasmonate compounds varies widely, ranging from 3 to 10 µg per 1 g of fresh weight [8,9]. More jasmonate compounds are present in the generative parts of the plant, i.e., the pericarp, fruit, and seeds, than in the vegetative parts, i.e., stems and leaves [8]. Biological and physicochemical factors as well as mechanical damage have a large influence on the increase in the amount of jasmonates [9,10]. Moreover, the amount of jasmonate compounds in the plant decreases with age (Figure 1) [8].

## 2. Chemical Structure of Jasmonate

The basic representative of the jasmonate group is jasmonic acid, i.e., 3-oxo-2-(pent-2′-enyl)cyclopentane acetic acid (IUPAC name), synthesized from linolenic acid found in the chloroplast membrane. Jasmonic acid (exhibiting optical activity) is composed of a cyclopentane ring to which three different substituents are attached, located at positions: C-3, C-6, and C-7 [11]. The following stereoisomers of jasmonic acid are known: *trans*-(-)-(3R,7R)-JA; (-)-JA; *trans*-(+)-(3S,7S)-JA; (+)-JA; *cis*-(-)-(3S,7R)-JA; (-)-*epi*-JA; *cis*-(+)-(3R,7S)-JA; (+)-*epi*-JA [12].

**Figure 2:**
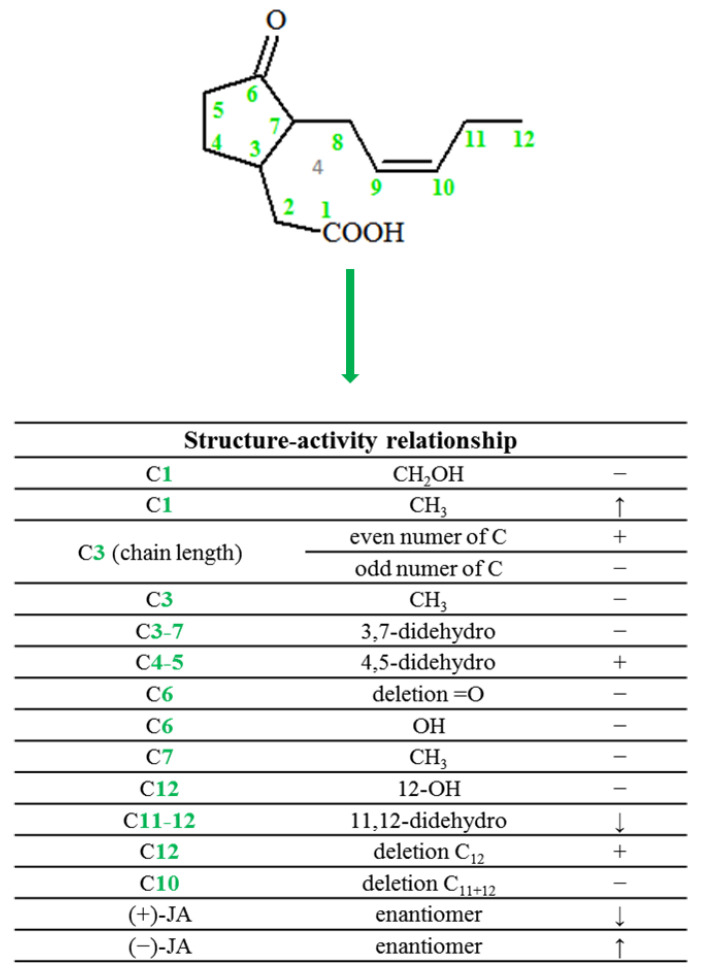
Structure-activity relationship of jasmonate compounds in relation to jasmonic acid. [↓, decreased activity; ↑, increased activity.; −, inactive; +, active].

In plants, jasmonic acid exists in the following forms: (-)-JA and (+)-*epi*-JA. Due to the fact that cis stereoisomers are thermodynamically less stable, they epimerize at the C-7 atom to the stable *trans* form, which at the same time shows higher biological activity (Figure 2). The structure of compounds and jasmonic acid derivatives requires the presence of a cyclopentanone ring with a ketone group at C-6. The substituent that significantly affects the activity is the pentenyl side chain at the C-7 position, while hydroxylation at carbon 11 or 12 as well as reduction between carbon 11 and 12 decrease the biological activity of the compound. When a chain with an odd number of carbon atoms is present at the C-3 position, a reduction of the activity of the compound has also been observed. Additionally, the activity of the compound is maintained or increased as a result of methylation or conjugation with amino acids [9,13,14,15].

Due to their structure, jasmonates tend to inhibit, induce, and stimulate changes that take place in plants at the morphological, cellular, and molecular levels.

## 3. Jasmonate Biosynthesis

Jasmonate biosynthesis in plants is analogous to the biosynthesis of eicosanoids in animal cells, with the difference that eicosanoids are synthesized from arachidonic acid (C20:4), while jasmonates are derived from α-linolenic acid (C18:3) (α-LA) released from chloroplast membranes by lipases (DAD1, DGL, PLA2) [16].

The JA biosynthetic pathway takes place in three parts of the cell: first in chloroplasts, then partially in peroxisomes, and finally in the cytoplasm. The first stage is the oxidation of α-linolenic acid in a reaction catalyzed by lipoxygenases (13-LOX; 9-LOX), which play a key role in the regulation of the synthesis of jasmonate compounds [17]. As a result of the synthesis with the participation of 13-LOX located in the inner membrane of chloroplasts, lipid hydroperoxides of 13S-hydroperoxyoctadecatrienoic acid (13-HPOT) are formed. These compounds are then metabolized by allene oxide synthases (13-AOS), (a family of oxygen-independent CYP74A and NADPH enzymes) to unstable allene oxides (12,13-EOT) [8]. In the course of successive transformations, allene oxide cyclase (AOC) catalyzes the transformation of allen oxide into *cis*-(+)-12-oxophytodienoic acid, which is the end product of a part of the jasmonic acid biosynthesis pathway in chloroplasts [18]. However, the exact release mechanism of 12-oxo-OPDA is not yet known. Currently, only one protein has been identified as the expression product of the COMATOSE gene, which due to its ATP transporter’s (ABC) activity and localization in peroxisomes is responsible for the transport of JA to the subcellular compartment in question [19,20].

**Figure 3:**
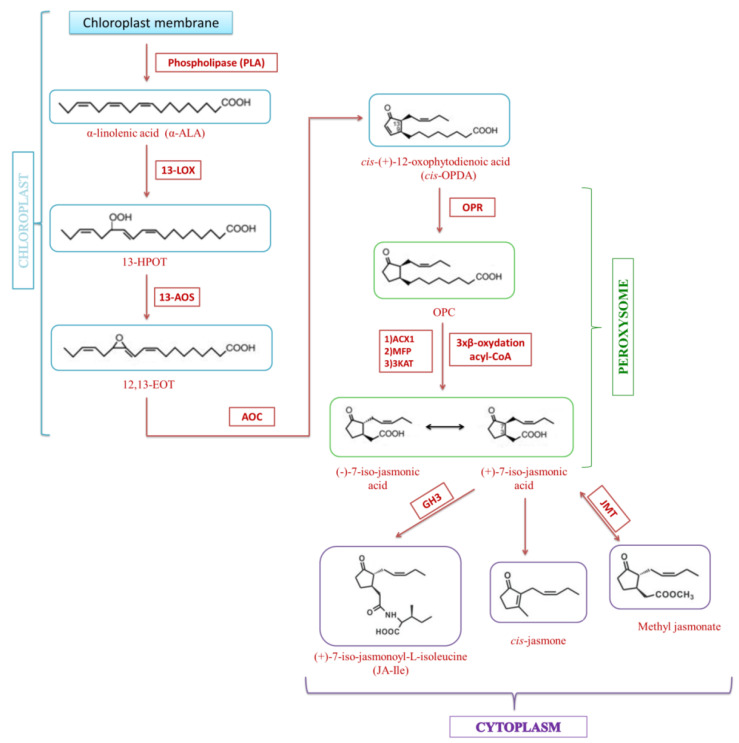
Synthesis of jasmonate compound. Abbreviations for compounds: α-ALA, α-linolenic acid; 13-LOX, 13-lipoxygenase; 13-HPOT,(13S)-hydroperoxyoctadecatrienoic acid; 13-AOS, allene oxide synthase; 12,13-EOT, 12,13-epoxyoctadeca-9,11,15-trienoic acid; AOC, allene oxide cyclase; JMT, jasmonate methyl transferase; GH3, Grechten Hagen 3; OPDA, cis-(+)-12-oxophytodienoic acid; OPR3, 12-oxo-phytodienic acid reductase3; OPC-8, 3-oxo-2-(2-pentenyl)-cyclopentane-1-octanoic acid; ACX1, acyl-CoA oxidase1; MFP, the multifunctional proteins; 3KAT, L-3-ketoacyl-CoA-thiolase; (3R,7R)(-)-JA, (3R,7R)(-) stereoisomer of jasmonic acid; (3R,7S)(+)-JA, (3R,7S)(+) stereoisomer of jasmonic acid.

In the next step, reduction of the cyclopentenone ring takes place, catalyzed by peroxisomic oxophytodienoic acid reductase (OPR) [21]. The remaining steps in the synthesis of jasmonic acid are already taking place in peroxisomes. They include three successive β-oxidation reactions with the participation of the appropriate acyl-CoA ester [22]. As a result of these reactions, the side chain is shortened and transformed into the pentenyl form. In the case of some plants, such as radish or tomato, three β-oxidative enzymes are responsible for this process, i.e., *acyl*-CoA oxidase (ACX1), multifunctional protein (MFP), and L-3-ketoacyl-CoA thiolase (KAT) [5]. The end product of the transformation of linolenic acid is 12-carbon (+)-7-isojasmonic acid, which after transport to the cytoplasm can easily transform into (-)-7-isojasmonic acid or undergo further metabolic transformations, including formation reactions of active or inactive derivatives with sugars or amino acids (Figure 3) [17]. Jasmonic acid is then transported to the cytoplasm where it can be modified by enzymes such as JAR1, IAR3, or ILL6 [23]. The best-described protein responsible for the formation of JA adducts with amino acids is GH3 (Grechten Hagen 3), which most often binds JA to isoleucine, thus creating a biologically active conjugate [24,25].

## 4. Metabolism of Jasmonate

**Figure 4:**
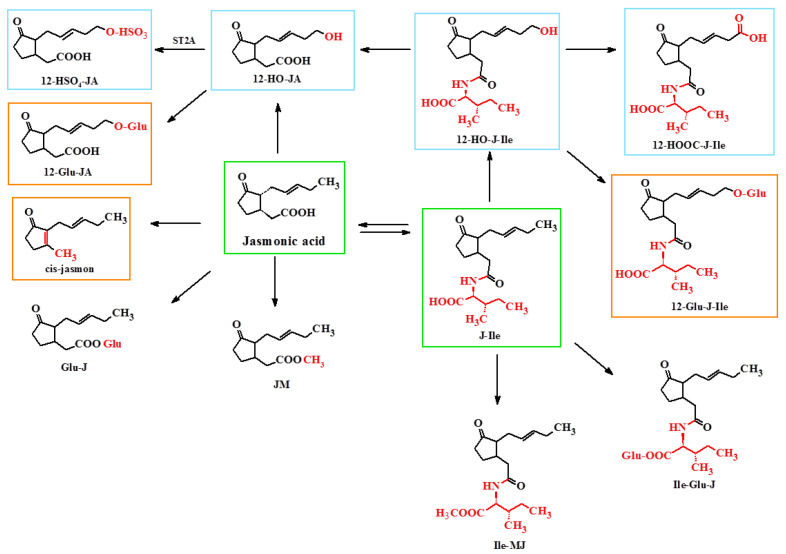
Jasmonic acid derivatives and metabolites; green box = derivatives active, blue box = derivatives inactive, orange box = derivatives partially active. 12-HSO3-JA, 12-bisulfate jasmonic acid; 12-HO-JA, 12-hydroxyjasmononic acid; 12-HO-JIle, (12-hydroxyjasmonoyl)isoleucine; 12-HOOC-J-Ile, (12-carboxyjasmonoyl) isoleucine; 12-Glu-JA, 12-glukozylojasmonic acid; J-Ile, jasmonoylisoleucine; 12-Glu-J-Ile, (12-glukosyljasmonoyl)isoleucine; Glu-J, glukosyl ester of jasmonic acid; JM, methyl ester of jasmonic acid; Ile-JM, methyl ester of jasmonoylisoleucine; Ile-Glu-J, glukosyl ester of jasmonoylisoleucine.

Currently, twelve metabolic pathways are known that convert jasmonic acid into active, partially active, or inactive compounds (Figure 4) [17]. Most of the compounds are derivatives of jasmonic acid and isoleucine, while some reactions lead to the formation of compounds that are active in specific reactions in response to stress and stressful situations, such as, for example, a leaf movement.

Metabolites of jasmonic acid include various compounds obtained as a result of the following: conjugation with isoleucine to obtain jasmonylisoleucine (JA-Ile) [10,26,27]; methylation to methyl jasmonate (MJ) [10]; ester formation with glucose to 12-glucosyljasmonic acid (12-Glu-JA) [28]; decarboxylation to *cis*-jasmon [29]. As a result of hydroxylation at the C-12 position, 12-hydroxyasmonic acid is formed (12-OH-JA), present in *Solanum tuberosum* [30]. However, as a result of the reduction of the ketone group at the C-6 position of jasmonic acid leading to the formation of a hydroxyl group, cucurbic acid is formed. It can undergo glycosylation, methylation, and hydroxylation. It has been found in the seeds of *Cucurbita pepo*, in the leaves of *Equisetum* sp., and in the stems of *Hordeum vulgare* [31]. Both JA stereoisomers, apart from hydroxylation, can undergo glycosylation (conjugation mainly with glucose and gentiobiose) and combine with amino acids occurring in the S configuration [10,31]. This pertains to aromatic amino acids (conjugates with phenylalanine, tryptophan, and tyrosine have been found in flowers) and aliphatic amino acids (conjugates with isoleucine have been found in leaves and fruits; with leucine and valine found in *Vicia faba* leaves). Less common is the conjugation of jasmonic acid with tyramine—a biogenic amine formed by the decarboxylation of tyrosine. This conjugate has been found in the petunia pollen. Moreover, jasmonates accumulating in tuber-producing plants (mainly tuberic acid) may undergo sulfonation [32].

## 5. The Role of Jasmonates in Plant Growth

Jasmonic acid has been found to be an inhibitor of pollen germination in plants such as *Camellia sinensis*, *Impatiens balsamina*, and *Lilium formosanum*. Jasmonic acid acts quite differently on seeds that are in a state of dormancy. It stimulates the germination of embryos isolated from maple and plane seeds. Exogenous jasmonic acid inhibits seedling elongation and limits the elongation of roots and stems. The roots are more sensitive to the action of jasmonates than the hypocotyl. Methyl jasmonate is presumed to prevent fruit softening by inhibiting the activity of peroxidase and polygalacturonase, which degrade cell wall polysaccharides. Methyl jasmonate also inhibits the activity of lipoxygenase, which results in an increase in the content of linolenic acid, tocopherols, and linoleic acid [33]. This phytohormone increases the content of plant pigments from the group of carotenoids in *Zea mays* corn kernels [34]. Exogenous methyl jasmonate reduces the level of lycopene and increases the amount of β-carotene in tomatoes [35]. In green fruit, jasmonate inhibits the accumulation of anthocyanins and accelerates the breakdown of chlorophyll [36]. Studies on *Oryza sativa* rice indicate that exogenous jasmonic acid regulates leaf aging by mediating chlorophyll and membrane degradation [37]. Research also indicates a possible participation of jasmonic acid in the fallout of *Glycine max* pods [38]. Moreover, methyl jasmonate probably stimulates leaf fall in *Vigna radiata* and *Kalanchoe blossfeldiana*, and influences the spring leaf fall of *Ficus superba* [39].

It has been found that the use of exogenous jasmonic acid may affect fertility in *Arabidopsis thaliana* mutants that exhibit male sterility, by not producing triene fatty acids. However, the precursor of jasmonates, i.e., 12-oxo-phytodienic acid, is necessary for the synthesis of jasmonates and required for the proper stamen and pollen development [19].

Jasmonates accumulate in injured tissues or cells of plants and function as the signal activating the expression of various genes, including defensins, i.e., proteins associated with resistance to pathogens, such as osmotines and thionines, or enzymes such as peroxidases, β-1,3-glucanases, and lipoxygenase [40]. They are also indicators or stimulators of the production of many secondary metabolites, i.e., phytoalexins, flavonoids, sesquiterpenoids, glucosinolates, or lignans, affecting the response to environmental stress [41].

Jasmonates are involved in the mycorrhiza. Treatment of *Avena sativum* with low concentrations of jasmonic acid stimulates the development of mycorrhiza, while high concentrations of jasmonic acid may increase the resistance of tissues that are sensitive to secondary pathogen-induced infections, drought, and osmotic stress. Spraying *Arabidopsis thaliana* with gaseous methyl jasmonate resulted in an effective reduction of the development of diseases caused by necrotic fungi *Alternalia brassicicola*, *Botrytis cinerea*, and *Plectosphaerella cucumerina*. Methyl jasmonate significantly reduced the accumulated amounts of sucrose as well as reducing sugars in the form of gums, in response to injury, pathogen or insect attack (Table 1) [42,43].

The presence of heavy metals in the environment affects the plant’s response, i.e., excessive production of jasmonates, and their accumulation is associated with the toxic mechanism of action of metals: limiting the growth of leaves and roots, accelerating the aging processes, and destroying the photosynthetic apparatus [44,45].

## 6. Similarities between the Action of Jasmonates on Plant and Animal Cells

There are certain similarities in the action of jasmonate compounds on plant and animal cells. Jasmonic acid leads to cell cycle arrest in the G2 phase of tobacco cells. This process results from the inhibition of the activity of proteins necessary for the initiation of mitosis (CDK B and cyclin B1) [28]. It also mediates in the programmed cell death in *Arabidopsis protoplasts* [60]. In the case of methyl jasmonate, it blocks the cell cycle in the G0-G1 phase in *Taxus cuspidate* cells in over 70%. [61]. On the other hand, rice cells exposed to jasmonate compounds exhibited a high activity of MAP kinases (OsMSRMK2, OsBIMK1, OsMSRMK3 oraz kinazy MAPK OsEDR1) [16], which are involved in the response to stress and pathogens. However, the action of jasmonates on neoplastic cells (MOLT-4; A549) activates some MAP kinases (c-Jun N-terminal kinase (JNK) i p38) [62].

It was found that methyl ester of jasmonic acid increases the level of ROS, thus leading to the death of some types of cancer cells [5]. Moreover, in plant cells, MeJA induces the production of hydrogen peroxide in a NAD(P)H dependent process [2]. In contrast, jasmonic acid leads to singlet oxygen-dependent death of *Arabidopsis thaliana* cells [63]. The increased level of ROS, induced by jasmonate compounds, is also responsible for the synthesis of heat shock proteins, both in plant and animal cells. It has been shown that methyl ester of jasmonic acid induces the synthesis of HSP72 protein in glioblastoma cells, in addition to activating the synthesis of low molecular weight HSP in *Douglas fir* seeds [16]. Jasmonates can induce similar metabolic, signaling, and stress-related modifications (which can lead to reduced growth and viability) in plant cells as well as in neoplastic animal cells. This suggests that as data concerning the effects of jasmonates in plant cells accumulates, it may provide predictive information that would prove necessary for the design of new anti-cancer drugs.

**Figure 5:**
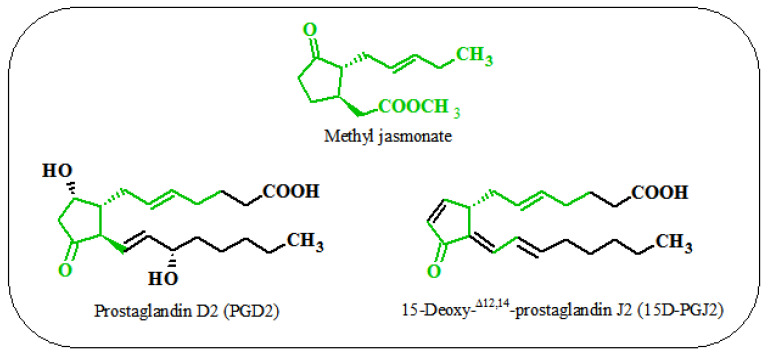
Similarities between jasmonates and prostaglandins.

Jasmonates are capable of inhibiting, inducing, or stimulating changes in plants at the morphological, physiological, cellular, and molecular levels. The most important reason for studying the biological activity of jasmonic acid and its derivatives in mammals, other than hormonal activity in plants, is the structural similarity of these compounds to prostaglandins, especially those exhibiting anti-inflammatory activity, including prostaglandin D2 (PGD2) and 15-deoxy-Δ12,14- prostaglandin J2 (15d-PGJ2) (Figure 5) [64].

## 7. Biological Activity of Jasmonates and Their Derivatives

### 7.1. Anti-Inflammatory

**Figure 6:**
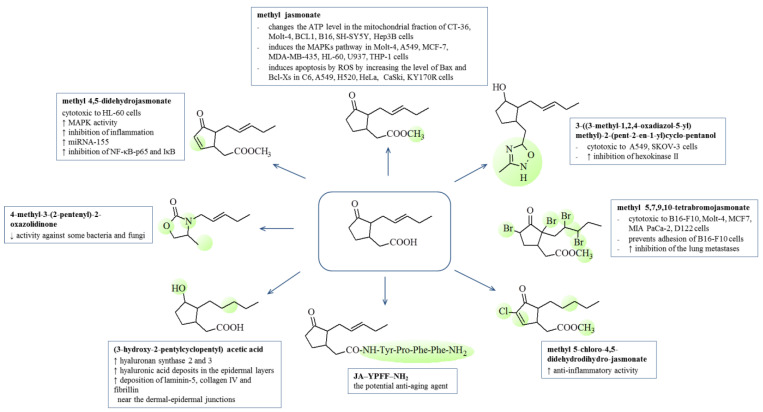
Biological activity of new jasmonate derivatives. [↓, decreased activity; ↑, increased activity].

The first studies on the potential anti-inflammatory activity of jasmonate compounds concerned MJ derived from *Gracilaria verrucosa* and showed that the effectiveness of MJ was comparable with or more effective than that of prostaglandin compounds [65]. The study demonstrated the inhibitory effect of MJ on the production of pro-inflammatory mediators (NO, IL-6 and TNF-α) in lipopolysaccharide activated RAW 264.7 mouse macrophages. However, the growing interest in jasmonic acid as a potential therapeutic agent led to the synthesis of new derivatives in order to obtain more active compounds. The basic structural modification changing the activity of jasmonates was the introduction of a double bond in the cyclopentyl ring. Chemically, α,β-unsaturated carbonyl compounds are electrophilic centers that are highly susceptible to addition reactions with nucleophiles such as free sulfhydryl groups of reduced glutathione or cysteine residues in proteins. Thus, prostaglandins are compounds in which cyclopentenone is the pharmacophore responsible for the biological activity of these compounds [66]. Therefore, the introduction of unsaturated bonds into the structure of methyl jasmonate with the formation of methyl 4,5-didehydrojasmonate (DHJM) resulted in the formation of compounds showing higher anti-inflammatory activity than similar prostaglandins [62]. Other modifications were the introduction of various halogens at the C3 position of α-cyclopentenone while maintaining the ring double bond and/or replacing the ester residue with an amide. It was found that the presence of a cyclopentenone fragment is necessary to maintain anti-inflammatory activity, while the presence of halogens increases this activity even with the simultaneous presence of saturated bonds in the side chain [65]. The α-haloenones formed in this manner also showed a higher anti-inflammatory activity than the natural prostaglandins. In 2012, Dang et al. synthesized a series of derivatives with various fragments of jasmonate esters, evaluated their resistance to hydrolysis and converted them into derivatives with a chlorine atom in the position of α-cyclopentenone [67]. The most active analogs in this series were t-butyl and hydroxyethyl esters, which was confirmed by the fact that the chain branching and the increased hydrophilicity in relation to the methyl moiety in MJ affect the anti-inflammatory activity (Table 2 and Figure 6).

### 7.2. Anticancer

Regardless of their anti-inflammatory properties, it has been shown that jasmonates have the properties characteristic for anticancer drugs. Their action is characterized by high selectivity in relation to neoplastic cells and effectiveness against neoplastic cells resistant to classic anticancer drugs. It has been found that jasmonic acid and jasmonate can increase the survival of animals and humans with lymphoma and induce apoptosis/necrosis of human cells of leukemia, lung, colon, prostate cancer, neuroblastoma, breast cancer, and melanoma, as well as chronic lymphocytic leukemia cells [71]. It is believed that there are three potential mechanisms of action of jasmonates: (i) bioenergy involving ATP depletion through mitochondrial disturbance, (ii) induction of redifferentiation by the activation of the MAPK kinase cascade, and (iii) induction of cell apoptosis by ROS generation (Table 3) [16].

Cellular ATP depletion induced by MJ is caused by a reduction of the interaction of hexokinase-2 enzyme with the VDAC (voltage-dependent anion-selective channel 1) protein, resulting in a decrease in the efficiency of glycolysis and, consequently, in the level of cellular ATP. Prolonged opening of VDAC channels changes the permeability of the mitochondrial membrane, disperses the membrane potential, increases osmotic edema, and releases pro-inflammatory factors, including cytochrome c, leading to cell death [5,16,72] As HK levels are up to 200 times higher in malignant tumor cells in comparison with normal cells [83], the enzyme is an excellent molecular target for potential anti-neoplastic agents. The specific binding of jasmonate to HK has been confirmed in an immunochemical test with the use of surface plasmon resonance and in tests of the VDAC activity of lipid bilayers without inhibiting kinase activity [73]. MJ has been shown to detach HK1 and HK2 from VDAC in a dose-dependent manner in the mitochondrial fraction of CT-26 murine colon carcinoma cells, MOLT-4 human leukemia, BCL1 murine leukemia, B16 murine melanoma, HCC hepatocellular carcinoma (LM3, BEL -7402, Hep3B, SMMC-7721), neuroblastoma (SH-SY5Y), B lymphoma, and liver cancer cells (Hep3B) [5,16,70,71,72,83]. The recently obtained synthetic derivative exhibiting hexokinase-2 inhibitory properties is 3-((3-methyl-1,2,4-oxadiazol-5-yl)methyl)-2-(pent-2-en-1-yl)cyclopentanol. The introduction of 1,2,4-oxodiazole in place of the methyl ester of jasmonate and the reduction of the ketone in cyclopentanone to the hydroxyl group resulted in an active inhibitor that showed cytotoxicity against the following human cell lines: A549 lung cancer and SKOV-3 ovarian cancer [70].

Methyl jasmonate has the ability to induce the Mitogen Activated Protein Kinases (MAPK) pathway, which determines the differentiation of neoplastic cells. Apoptotic cell death has been observed in MOLT-4 leukemia and A549 lung cancer cells treated with MJ by releasing the JNK and p38 proteins, resulting in the activation of the transcription factor AP-1 [75]. Although AP-1 is a protein involved in both apoptosis and differentiation, no effect of AP-1 on apoptosis was found in MOLT-4 and A549 cells. In both MCF-7 (receptor-dependent) and MDA-MB-435 (receptor-independent) breast cancer cell types, methyl jasmonate caused apoptosis but activated the p38 and ERK pathways via MAPK only in receptor-independent cells [76,78]. MJ activated the MAPK pathway in the HL-60 human myelocytic leukemia cell line, which instead of apoptosis caused the cells to differentiate into monocyte-like granulocytes [79]. In the same way, MJ also acts on U937 histiocytic lymphoma and THP-1 acute monocytic leukemia cells, which are the model cells for studying the behavior and differentiation of monocytes [16]. MJ has been shown to inhibit the growth of leukemic blasts and stimulate their differentiation into normal cells as evidenced by the expression of differentiation markers such as NBT reduction of nitrotetrazolium blue (for myelomonocyte differentiation), morphological differentiation into granulocytes, and the expression of CD14 cell differentiation antigens (specific for monocytes) and CD15 (granulocyte-specific) [62].

Another antitumor mechanism of action of jasmonic acid and its methyl ester is the increase in the level of ROS in the following cells: glioblastoma (C6), non-small cell lung cancer (A549 and H520), and uterine cancer (HeLa and CaSki) [76,80,81,82,84,85,86]. In human non-small lung cancer cells (A549) the above compounds increase the expression of pro-apoptotic proteins from the Bcl-2, Bcl-Bax and Xs families [87], while in prostate cancer cells PC-3 increase the expression of the anti-apoptotic protein Bcl-2 [88]. This causes the cell cycle to arrest in the G2-M phase and to activate the executor caspase, then directing cells to apoptosis (Figure 6).

**Figure 7:**
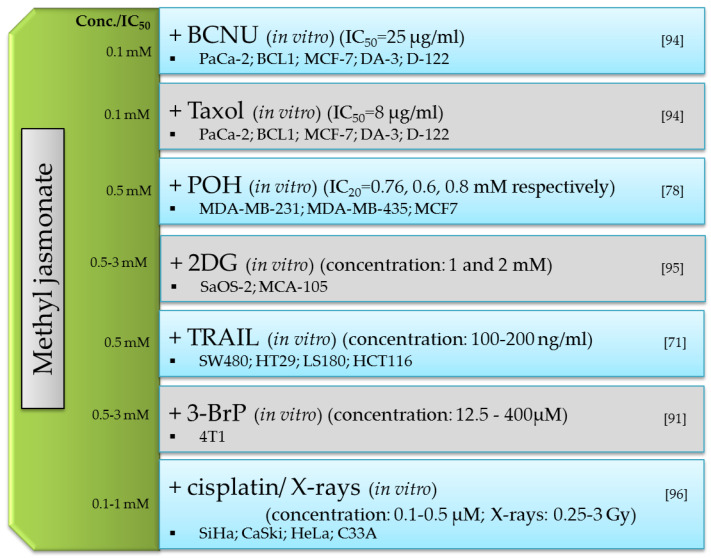
Therapeutic effects of combining MJ with anticancer compounds.

In addition to the fact that jasmonates themselves induce apoptosis in tumor cells, they can also be combined with other anti-tumor agents to achieve synergistic effects. Therefore, studies have been conducted to evaluate the effects of combining MJ with various anticancer compounds [89], that are routinely used in clinical practice: BCNU (carmustine), cisplatin, paclitaxel (taxol) [90], doxorubicin (adriamycin), and 3-bromopyruvate (3-BrP) (Figure 7) [91]. Due to the fact that the target organelles for these drugs are mitochondria, although they act according to different mechanisms, the use of MJ may result in additive efficacy. The use of BCNU alone induces mitochondrial DNA damage [92], while cisplatin and taxol induce mitochondrial membrane depolarization and cytochrome c release [93]. Interactions of these compounds with MJ have been observed in many cell lines of malignant neoplasms, such as: breast, lung, prostate, and pancreatic cancer, as well as leukemia [91]. MJ drastically lowered the IC_50_ values of the drugs used, at the same time reducing the side effects of these drugs [5]. It has also been found that inhibition of glycolysis by 2DG in 29M4.1 B lymphoma cells enhanced the effect of MJ by drastically reducing cellular ATP levels, this effect was much more potent than the effect induced by MJ alone [70]. The cytotoxic effects of the combination of MJ (1 mM) and 2DG (5 mM) have also been confirmed in the following tumor cells: CT26, D122 and MCF-7 [94,95]. The use of MJ with cyclophosphamide as a routine treatment for breast cancer in tumor-bearing mice resulted in a reduction in tumor volume and an increase in tumor growth inhibition. Moreover, the applied therapy had no significant side effects on kidneys, liver, the immune system, and body weight [91].

Recently, it has also been shown that MJ effectively interacts with cisplatin and radiotherapy in the treatment of cervical cancer cells by significantly reducing the doses of radiation and cisplatin required to inhibit cell survival [5,96]. The study showed for the first time that alpha-radiation selectively reduces cell viability and cervical cancer cell survival.

### 7.3. Cosmetic Activities

Methyl jasmonate is a fragrance ingredient used in many fragrance products. It is a colorless oily liquid with a strong sweet floral-herbal odor, representing the typical background notes of jasmine absolute. The smell of jasmine oil is largely due to (1R,2S)-(+)-methyl Z-epijasmonate, which is one of the main components of the oil obtained from jasmine flowers and constitutes 2–3% of the weight of its composition. It can be found in fragrances used in cosmetics, perfumes, shampoos, and soaps, as well as in non-cosmetic products such as cleaners and detergents [8,97]. Studies on albino rabbits and guinea pigs which were topically administered methyl jasmonate (2 g/kg and 10% MJ solution, respectively) showed that it did not irritate the skin. At a concentration of 10%, it also did not irritate human skin, which was confirmed by a 3-week study on 50 volunteers [98].

Regardless of the above, jasmonates have also been used in cosmetic agents responsible for soothing skin irritations, stimulating the exfoliation and renewal of the epidermis, and demonstrating the ability to regulate the activity of the sebaceous glands in the skin [97]. A new derivative of jasmonic acid, i.e., tetrahydrojasmonic acid (trade name LR2412) has been used, among others, in skin care cosmetics. The effect of LR2412, (1 and 10 µm) was investigated on an in vitro reconstructed skin model, Episkin^TM^. LR2412 has an anti-aging effect because it stimulates the synthesis of the following enzymes involved in the production of acid: hyaluronan synthase 2 and hyaluronate synthase 3. As a consequence, the following were observed: an increase in hyaluronic acid deposits in the basal and suprabasal layers of the epidermis, stimulation of the deposition of laminin-5, collagen IV, and fibrillin near the dermal-epidermal junctions. It was found that a tetrahydrojasmonic acid molecule has the ability to penetrate the epidermis and the upper layers of the dermis. The research results presented by Bouloc et al. prove that tetrahydrojasmonic acid in combination with retinol yields good results in the treatment of the signs of photoaging. It was also found that the composition containing 0.2% of retinol and 2% of LR2412 is better tolerated by the skin of patients compared to the preparation containing only a 0.025% of tretinoin [99].

The N-terminal conjugate of jasmonic acid with the YPFF-NH2 peptide may also have a potential cosmetic application, but apart from the synthesis, no biological research is available yet [97]. Commercially available acetyl-YPFF tetrapeptide, when applied to the skin, weakens the stimulation of nerve endings, resulting in a reduction of skin hypersensitivity, while jasmonic acid stimulates the renewal of the epidermis. The authors suggest that this combination may be responsible for the reduction of the visible effects of aging by the peptide derivative of jasmonic acid.

Jasmonate derivatives containing heteroatoms in the cyclopentyl ring (N, O) in place of some carbon atoms have been tested for their odor properties. The unsaturated *cis* double bond, present on the alkyl side chain at position 2 in natural *cis*-jasmone, plays a very important role as far as the fragrance properties are concerned, and the introduction of the double bond into the heterocyclic jasmone analogs, both *cis*- and *trans*-, leads to an increase in odor intensity relative to their saturated heteroanalogs [100]. These derivatives have also been tested (e.g., 4-methyl-3-(2-pentenyl)-2-oxazolidinone) as potential antimicrobial compounds (*Staphylococcus aureus MRSA*, *Enterococcus faecalis*, *Klebsiella pneumoniae*, *Escherichia coli*, *Salmonella enteritidis*, *Pseudomonas aeruginosa*, *Candida albicans*, *Aspergillus flavus*, and *Microsporum gypseum*), but unfortunately all of them showed less activity than the parent jasmonates (Figure 6) [101].

## 8. In Vivo Effect of Methyl Jasmonate and Synthetic Tetrabromojasmonate

Methyl jasmonate is considered safe for humans based on the widespread consumption of plants. There are no reports on the toxicity of this substance by any route of exposure and it has been recognized by the Food and Agriculture/World Health Organization (FAO/WHO) as a food additive in the group of flavoring agents [102]. Due to the number of in vitro tests confirming the fact that MJ does not have cytotoxic effect on healthy cells, only a few in vivo tests are known (Table 4).

One of the first reports of in vivo studies concerned methyl jasmonate administered to C57BL/6J mice infected with EL-4 leukemia cells. Their survival time was significantly longer compared to mice not treated with jasmonate [103].

Subsequent studies focused on the effects of MJ and its synthetic derivative methyl 5,7,9,10-tetrabromojasmonate (TBMJ), on lung metastases in C57BL mice injected with B16-F10 melanoma cells, at doses of 40 and 75 mg/kg MJ and 20 mg/kg TBMJ of body weight respectively, which were considered non-toxic [69]. The synthetic compound proved to be significantly more potent than MJ in vivo as it generated a similar inhibitory effect on tumor growth at doses per mole that were approximately nine times lower.

Lopes et al. confirmed that MJ in concentrations of 1–10 µM inhibits angiogenesis in the chorioallantoic (CAM) membrane of the chicken embryo [104]. Methyl jasmonate was applied topically to neoplastic and pre-cancerous lesions in eight patients, in the form of an oily solution containing 1 mg of the substance per 1 mL of solution [105]. It did not cause any significant local or systemic side effects. Three patients showed positive responses. Two patients recovered completely and one relapsed three months after treatment.

The potential of MJ as a new therapeutic agent against multiple myeloma in vivo was also considered [106]. Mice were injected with MM.1S cells and treated with 1000 mg MJ/kg body weight for 8 weeks. Tumor growth was monitored by physical parameters (weight loss, apparent tumor weights, hind limb paralysis) and overall survival was taken as the study endpoint. After 150 days, 15/15 control mice treated with the carrier only died, while 8/15 MJ-treated mice survived.

To confirm the protective role of MJ in lipopolysaccharide-induced arthritis in Wistar rats, MJ was administered intraplantally at two doses (20, 40 mg/kg), with indomethacin as the standard. By behavioral evaluation of thermal hyperalgesia of the paw and tail, MJ was found to reduce hyperalgesia to hot and cold stimuli as a withdrawal latency [107].

In the mouse thymus-derived tumor called Dalton’s Lymphoma (DL) [108] investigated whether intra-tumor administration of MJ may result in an increase of tumor growth impedance. The result was a delayed tumor growth associated with prolonged survival of the tumor bearing mice. The MJ-dependent tumor growth delay was associated with a decrease in the blood supply to the tumor environment, cell cycle arrest, increased induction of apoptosis and necrosis, deregulation of glucose and lipid metabolism, increased fragility of cancer cell membranes, and altered cytokine composition in the tumor.

## 9. Conclusions

Due to the synergistic activity of substances isolated from plants, herbal medicines have a fairly wide range of action, but the isolated individual substances may have different effects. Jasmonic acid, as well as its natural and synthetic derivatives, is one of the few examples of a single compound of natural origin that shows a wide range of activity. The presented paper aims to identify jasmonates as potential therapeutics.

The interest in jasmonates and their derivatives stems from their structural similarity to prostaglandins, and this feature shows potential for the use of this compound as a therapeutic agent. Methyl jasmonate and other new derivatives show a higher anti-inflammatory activity than natural prostaglandins. Jasmonates exhibit the perfect characteristics of anti-cancer agents due to the fact that they act on cancer cells but not on healthy cells. At the same time, they perfectly interact with known chemotherapeutic agents, enhancing their effect. Jasmonates and their derivatives inhibit in vitro proliferation of neoplastic cells such as: myeloid leukemia, breast, prostate, melanoma, stomach, cervix, colon, large intestine, hepatoma, lung, neuroblastoma, sarcoma, and lymphoblastic leukemia. The mechanisms explaining the anti-cancer activity of jasmon compounds proposed are based on: changes in the ATP levels in the cell; induction of redifferentiation through MAPK activity; and ROS-induced apoptosis.

Unfortunately, so far there have been no documented clinical trials focused on jasmonates as therapeutics, apart from patents that report their potential use. However, it must be remembered that some diseases result from the impairment of those mechanisms that are responsible for the beneficial effects of jasmonates on cancer cells. ROS production, widely reported in the literature, may be one such example. The mechanism involving the stimulation of ROS production by jasmonates, resulting in apoptosis of neoplastic cells, may be the cause of the intensification of oxidative stress affecting the development of other diseases.

In the authors’ opinion, it is too early to discuss the effects of jasmonates on the whole body, due to the fact that the therapeutic doses of jasmonates–and thus their side effects–are not yet known. Since jasmonate works according to various mechanisms, it is uncertain whether these will cause treatment-excluding effects in various, often comorbid, diseases.

## Figures and Tables

**Table 1:** Action of jasmonates in plants.

Activity	Plant	Action of Jasmonates	Literature
Germination	*Pinus nigra* *Prunus armeniaca*	inhibits pollen germination	[46,47]
Plant growth	*Hordeum vulgare* *Avena sativa* *Triticum aestivum* *Helianthus annuus*	inhibits the elongation of seedlings limits elongation of roots and stems	[48,49,50,51,52]
Ripening fruit	*Fragaria chiloensis*	prevents softening of the fruit	[53]
Aging of leaves	*Oryza sativa* *Hordeum vulgare*	accelerates the breakdown of photosynthetic pigments	[54,55]
Organ prolapse	*Arabidopsis Rubisco*	accelerates the fall off of pods and leaves	[56]
Male organ fertility in higher plants	*Arabidopsis thaliana*	reduces male fertility	[57]
Overall development	*Medicago sativa*	lengthens shoots slows down the induction of the embryo somatic differentiation process	[58]
Metabolic processes	*Avena sativum* *Solanum lycopersicum*	low concentration-stimulates the development of mycorrhiza high concentration-reduces the mycorrhization capacity of the roots	[42,43]
Formation of tubers and roots	*Arabidopsis thaliana*	increases the weight of tubers induces the formation of lateral roots	[59]

**Table 2:** New jasmonate derivatives with anti-inflammatory activity.

Jasmonates Derivatives	Cells	Concentration/IC_50_	References
Methyl jasmonate	RAW264.7 (macrophage)	50 and 100 μM	[65]
methyl 4,5-didehydrojasmonate DHJM	RAW264.7 (macrophage)	6.25, 12.5, 25, and 50 μM	[68]
methyl 5,7,9,10-tetrabromojasmonate	melanoma cells B16-F10	0.042 mM	[69]
methyl 5-chloro-4,5-didehydrodihydro-jasmonate	RAW264.7 (macrophage)	12.5 and 20 µM	[65]
t-butyl 5-chloro-4,5-didehydrodihydro-jasmonate	RAW264.7 (macrophage)	3.12, 6.25, 12.5 and 25 μM	[67]
3-((3-methyl-1,2,4-oxadiazol-5-yl) methyl)-2-(pent-2-en-1-yl)cyclo-pentanol	A549 SKOV-3	4564 mM 6077 mM	[70]

**Table 3:** Anticancer mechanism of MJ action.

MJ-Mechanism of Anticancer Action	Cancer Cells	MJ IC_50_	Literature
	lymphoma B	2 mM	[72]
bioenergy involving ATP depletion via mitochondrial disturbance	mouse colon cancer CT-26	3 mM (max conc)	[73]
	human T-lymphoblastic leucemia cell line MOLT-4	3 mM (max conc)	[73]
	mouse leucemia BCL1	3 mM (max conc)	[73]
	mouse melanoma B16	2.6 mM	[69]
	hepatocellular carcinoma HCC (LM3, BEL-7402, Hep3B, SMMC-7721)	1.65 mM	[5]
	neuroblastoma SH-SY5Y	3 mM (max conc)	[74]
	liver cancer Hep3B	0.5 mM	[75]
induction of re-differentiation by activation of the MAPK kinase cascade	human T-lymphoblastic leucemia cell line MOLT-4	0.5 mM	[75]
	lung cancer A549	4.937 mM	[76]
	human breast cancer MCF-7	2 mM	[77]
	human melanocytic MDA-MB-435	1.9 mM	[78]
	leukemia HL-60	0.4 mM	[79]
induction of apoptosis by the generation of ROS	glioblastoma C6	5 mM	[80]
	non-small cell lung cancer A549 i H520	2 mM and 2.5 mM	[78]
	cervical carcinoma HeLa, CaSki, SiHa i C33A	3.0 mM, 2.2 mM, 3.3 mM and 1.7 mM	[81]
	prostate cancer PC-3	5 mM	[82]

**Table 4:** In vivo effect of methyl jasmonate and synthetic tetrabromojasmonate.

Jasmonates	Concentration/ Exposure Time	Organism	Effects	References
**MJ**	0.5–3 mM, 24 h	C57BL/6 mice bearing EL-4 lymphoma	↑ survival time compared to untreated control	[105]
**MJ** **methyl 5,7,9,10-tetrabromojasmonate (synthetic)**	40 or 75 mg/kg body weight, 5 days a week, 3 weeks 20 mg/kg body weight, 5 days a week, 3 weeks	B16-F10 cells inoculated i.v. into the tail vein of C57BL mice to produce tumor growth in lungs	↓ lung metastasis	[70]
**MJ**	1–10 µM, 5 days	chorioallantoic (CAM) membrane of the chicken embryo	↓ angiogenesis	[106]
**MJ**	1 mg/1 mL, twice daily on the diseased skin or mucus for 4 weeks	pre-malignant and malignant skin lesions in 8 patients in the 56–73 age range	3 patients showed positive responses 2 patients recovered 1 patient relapse three months after treatment	[107]
**MJ**	1000 mg/kg body weight, 5 days a week over the next 8 weeks,	multiple myeloma in mice	overall survival for 150 days after MM cell injection	[108]
**MJ**	20 and 40 mg/kg body weight	Wistar rats	↓ lipopolysaccharide induced arthritis in rats	[107]
**MJ**	intra-tumoral administration	murine thymus-derived tumor Dalton’s Lymphoma	↓tumor growth ↑survival of the tumor-bearing mice	[108]

[↓, decreased activity; ↑, increased activity].
